# Urinary Mitochondrial DNA Identifies Renal Dysfunction and Mitochondrial Damage in Sepsis-Induced Acute Kidney Injury

**DOI:** 10.1155/2018/8074936

**Published:** 2018-02-26

**Authors:** Qiongyuan Hu, Jianan Ren, Huajian Ren, Jie Wu, Xiuwen Wu, Song Liu, Gefei Wang, Guosheng Gu, Kun Guo, Jieshou Li

**Affiliations:** ^1^Department of Surgery, Jinling Hospital, Nanjing University Medical School, Nanjing, China; ^2^Department of General Surgery, Nanjing Drum Tower Hospital, The Affiliated Hospital of Nanjing University Medical School, Nanjing, China

## Abstract

**Background:**

Recent animal studies have shown that mitochondrial dysfunction initiates and accelerates renal injury in sepsis, but its role in sepsis remains unknown. Mitochondrial stress or dying cells can lead to fragmentation of the mitochondrial genome, which is considered a surrogate marker of mitochondrial dysfunction. Therefore, we evaluated the efficiency of urinary mitochondrial DNA (UmtDNA) as a marker of renal dysfunction during sepsis-induced acute kidney injury (AKI).

**Methods:**

We isolated DNA from plasma and urine of patients. mtDNA levels were quantified by quantitative PCR. Sepsis patients were divided into no AKI, mild AKI, and severe AKI groups according to RIFLE criteria. Additionally, cecal ligation and puncture (CLP) was established in rats to evaluate the association between UmtDNA and mitochondrial function.

**Results:**

A total of 52 (49.5%) developed AKI among enrolled sepsis patients. Increased systemic mtDNA did not correlate with systemic inflammation or acute renal dysfunction in sepsis patients, while AKI did not have an additional effect on circulating mtDNA levels. In contrast, UmtDNA was significantly enriched in severe AKI patients compared with that in the mild AKI or no AKI group, positively correlated with plasma creatinine, urinary neutrophil gelatinase-associated lipocalin, and kidney injury molecule-1, and inversely with the estimated glomerular filtration rate. Additionally, UmtDNA increased in rats following CLP-induced sepsis. UmtDNA was predictive of AKI development and correlated with plasma creatinine and blood urea nitrogen in the rat sepsis model. Finally, the UmtDNA level was inversely correlated with the cortical mtDNA copy number and relative expression of mitochondrial gene in the kidney.

**Conclusion:**

An elevated UmtDNA level correlates with mitochondrial dysfunction and renal injury in sepsis patients, indicating renal mitochondrial injury induced by sepsis. Therefore, UmtDNA may be regarded as a valuable biomarker for the occurrence of AKI and the development of mitochondria-targeted therapies following sepsis-induced AKI.

## 1. Introduction

Sepsis is an intricate clinical condition characterized by stimulation of the systemic inflammatory response related to infection [1]. Acute kidney injury (AKI) occurs frequently in sepsis patients and is linked to high morbidity and mortality [2]. Patients suffering from AKI present an increased risk of progression to chronic kidney disease (CKD) and end-stage renal disease and have a major impact on healthcare resources [3]. Thus, a clear clarification of the pathogenesis responsible during renal dysfunction induced by sepsis is critical to avoid these profound complications and develop novel therapies to treat this prevalent disease.

Recent studies have implicated mitochondrial dysfunction to play a causative role in the pathogenesis of AKI following sepsis. Disruption of mitochondrial integrity in renal tubular cells seems to be a hallmark for diverse forms of AKI [4]. In sepsis-induced AKI mice, decreased antioxidant defenses and injured mitochondrial respiration coexist with the intrarenal inflammatory response and oxidative stress [5, 6]. Additionally, downregulation of protein expression during mitochondrial metabolism and decreased oxygen utilization have been observed in experimental models of sepsis-associated AKI [7]. Previous studies have also demonstrated that mitochondria-targeted drugs protected against abnormal mitochondrial structures and dysfunction in sepsis-induced AKI [8]. Similarly, the reversion of mitochondrial damage is able to prevent tubular cell apoptosis and oxidative stress in septic rats [9]. However, whether renal dysfunction is mediated by mitochondrial injury after sepsis remains unknown, partly owing to the lack of practical methods to detect mitochondrial damage and dysfunction. Moreover, human data are limited because renal biopsy is unlikely to be performed in AKI patients following sepsis.

Elevated mitochondrial DNA (mtDNA) levels in the urine has been considered as a novel noninvasive biomarker for detecting mitochondrial dysfunction. Eirin et al. revealed that increased urinary mtDNA (UmtDNA) in hypertensive patients correlated with other biomarkers of renal dysfunction and glomerular hyperfiltration [10, 11]. Likewise, we have recently showed UmtDNA is elevated in critical patients with AKI and associated with progression in severity [12]. However, whether derangements of mitochondrial integrity may be associated with the detectable release of UmtDNA in sepsis-induced AKI has never been determined. Therefore, we conducted experiments to test the effectiveness of UmtDNA as a predictor of AKI development and exacerbation induced by sepsis. In addition, we confirmed the effectiveness of UmtDNA as evidence of renal mitochondrial integrality in a rat model of AKI following sepsis.

## 2. Materials and Methods

### 2.1. Ethical Considerations

This study has been approved by the Institutional Review Board of Jinling Hospital, Nanjing (2016NZGKJ-061), and a written informed consent was obtained from each enrolled participant.

### 2.2. Patients and Sample Preparation

Human urine samples were obtained from 105 sepsis patients who were consecutively admitted to the surgical intensive care unit (SICU) at Jinling Hospital from June 2016 to July 2017. The SICU belongs to the department of general surgery, which is primarily for trauma and postoperative patients. Urine and plasma were collected within 3 hours in no AKI and AKI patients following the diagnosis of sepsis by a physician. Exclusion criteria were as follows: (a) refused consent; (b) patients with end-stage renal disease or chronic renal failure; (c) patients already on renal replacement therapy (RRT); (d) patients who had a history of acute coronary disease and stroke; and (e) patients who had previous renal transplant. Renal function was evaluated by plasma creatinine and BUN, and patients were stratified according to the RIFLE criteria [13]. In the present study, participants were subdivided into 3 groups in accordance with changes in RIFLE stage to assess the UmtDNA diagnostic efficacy, including no AKI, mild AKI (risk of renal failure), and severe AKI (injury to the kidney, failure of kidney function, or the need for RRT). Following urinary collection, protease inhibitors were added to the urine followed by centrifugation at 1000 ×g for 3 min. After removal of intact cells and cellular debris, the supernatants were harvested and stored at −80°C prior to DNA extraction, as previously described [12, 14].

### 2.3. Isolation and Quantification of mtDNA

In the present study, free DNA was isolated from 200 *μ*L plasma using the QIAamp DNA Blood Mini Kit (Qiagen, Valencia, CA), and DNA was also extracted from 1.7 mL urine using the urine DNA isolation kit from Norgen Biotek (Ontario, Canada; catalog number 18100) as previously described [12, 15]. Each DNA concentration was measured by NanoDrop spectrophotometer. 10 ng DNA from each human urine was put into polymerase chain reaction (PCR), and 0.5 ng DNA was isolated in rat urine or kidney for the PCR reactions. Quantitative PCR targeting the mitochondrial gene (*COX3* and *ND1*) and the nuclear gene GAPDH was applied to quantify UmtDNA levels. For human urine, a standard curve by total DNA extracted from HK2 cells was established to calculate absolute values of mtDNA and nuclear DNA (nDNA). For rat urine, threshold cycle (Tc) helped to represent the relative abundances of UmtDNA because of undetectable levels of urinary glyceraldehyde phosphate dehydrogenase (GAPDH). The final reaction volume was 20 *μ*L. The thermal profile was set up as follows: 50°C for 2 minutes, 95°C for10 minutes, 40 cycles of 95°C for 15 seconds, and 60°C for 1 minute. Of note, higher Tc represents lower levels of UmtDNA DAMPs, as we previously described [15].

### 2.4. Renal Injury Markers

Urinary neutrophil gelatinase-associated lipocalin (NGAL) and kidney injury molecule-1 (KIM-1) were chosen as renal injury markers for sepsis patients. They were measured by ELISA according to a standard protocol (EK-Bioscience, EK-H12141, Shanghai, China, and EK-Bioscience, EK-H11514, Shanghai, China, resp.), as previously described [10].

### 2.5. Rat Cecal Ligation and Puncture Model

The 8- to 10-week-old male Sprague-Dawley rats (180–200 g) were subjected to sham or cecal ligation and puncture (CLP) surgery as described previously [16]. The rats were reared under standard laboratory conditions and had free access to food and water but fasted overnight prior to the experiments. The rats were kept in metabolism cages to collect urine after CLP surgery. Urine was collected from 6 to 24 h after surgery. The rats were euthanized with CO_2_ gas at 24 h, and blood and kidneys were collected. Renal function was analyzed by measuring the blood urea nitrogen (BUN) and serum creatinine levels using specific commercially available kits: BUN Assay Kit (Jiancheng Biotech, Nanjing, P. R. China) and Creatinine Assay Kit (Biosino Bio-Technology). Ethical approval was obtained from the local research ethics committee of Jinling Hospital.

### 2.6. Histology and Tubular Injury Score

The renal tissues of the rats were stained by H&E and observed under microscopy (400x) to score the pathologic injury of the renal tissue. The pathological changes in the kidney tissue were examined by light microscopy, and the slices were reviewed in a blinded manner and scored with a semi-quantitative scale to evaluate changes. Tubules that demonstrated cellular necrosis, vacuolization, cast formation, loss of the brush border, and tubule dilation were scored as follows: 0, none; 1, <11%; 2, 11% to 25%; 3, 26% to 45%; 4, 46% to 75%; and 5, >75%.

### 2.7. mtDNA Copy Number and mRNA Transcription Levels

For quantification of the mtDNA copy number, total DNA was extracted using the DNeasy Tissue Kit (Qiagen, Valencia, CA). We compared the relative amounts of mtDNA and nuclear DNA contents. The mtDNA amplicons were generated from a complex IV segment, and the nuclear amplicons were generated through amplification of a GAPDH segment. Tc values of mtDNA and GAPDH were determined for each individual quantitative PCR run. The ddCt (mtDNA to GAPDH) represented the mtDNA copy number in a cell. The following primers were used to determine mRNA expression levels for mitochondrial gene expression: PGC-1*α* and NDUFB8. Primers for qPCR analyses of the relevant sequences are listed in Supplementary Table 1. GAPDH was selected as the internal standard.

### 2.8. ATP Content Determination

Adenosine triphosphate (ATP) Determination Kit (Beyotime) was used to detect ATP concentration according to the manufacturer's instruction. ATP concentration was calculated with the construction of a standard ATP calibration curve.

### 2.9. Statistical Analysis

SPSS 19.0 statistical software (SPSS Inc. Chicago, IL) was used to perform statistical analyses. Data were expressed as the means ± SD or median ± IQR for continuous data and as proportions for categorical data. Univariate analyses were conducted using Student's *t*-test for continuous data and *χ* ^2^ test or Fisher's exact test for categorical variables. Receiver operating characteristic (ROC) curve was drawn to evaluate the predictive effectiveness of UmtDNA for AKI progression. The optimal cutoff point was calculated by determining the index that provided the greatest sum of sensitivity and specificity. Multiple variables underwent correlation, and their degree of correlation was performed by calculating Pearson correlation coefficients. The criterion for statistical significance was *P* < 0.05 for all comparisons.

## 3. Results

### 3.1. Patient Demographics

**Figure 1:**
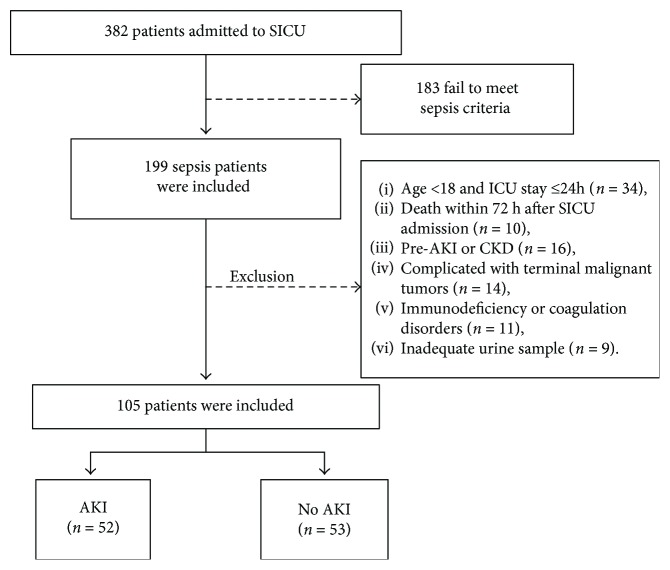
Flow chart of the septic patients with no AKI or AKI in this study. SICU: surgical intensive care; AKI: acute kidney injury; CKD: chronic kidney disease.

Consecutive critically ill patients who were admitted to the SICU from June 2016 to July 2017 were screened for this study. Among them, 105 patients who met our criteria were eligible and finally enrolled (Figure 1). The cohort demographics are summarized in Table 1. These 105 patients included 82 men and 23 women, with an average age of 51.3 year. During the first 7 days after admission, 52 patients developed AKI, whereas the other 53 patients had no AKI. There was no statistical significance between their underlying diseases and vital signs at admission. However, septic AKI patients were more likely to experience septic shock and mechanical ventilation after admission (*P* = 0.034 and 0.037, resp.), and their APACHE II and SOFA scores were significantly higher than those patients without AKI (*P* = 0.001 and 0.006, resp.). Although hospital mortality was higher in patients with AKI compared with that in the non-AKI group, the difference did not achieve statistical significance. Furthermore, Table 1 shows that urinary NGAL (*P* < 0.001) and KIM-1 (*P* = 0.001) were significantly elevated in AKI group compared with those in the non-AKI group.

### 3.2. Increased Circulating mtDNA Does Not Correlate with Biomarkers of Systemic Inflammation and Renal Function in Septic Patients

To analyze whether AKI was associated with enhanced circulating mtDNA, the levels of systemic mtDNA in septic patients were measured by qPCR. We previously showed that the plasma mtDNA level was increased in septic patients compared with that in critically ill patients without sepsis [15]. Although plasma mtDNA levels were elevated in septic patients with AKI compared with those in septic patients without AKI, no significant difference was found between the two groups (Supplementary Figure 1). To investigate whether this increase was associated with an increased inflammatory response and renal dysfunction, we performed correlation analysis between circulating mtDNA and leukocyte counts, plasma CRP, PCT, IL-6, creatinine, urinary NGAL, and KIM-1.  Table 2 shows that no significant correlation could be detected between circulating mtDNA levels and these indexes.

### 3.3. Urinary mtDNA Is Associated with Sepsis-Induced AKI Progression in Severity

**Figure 2:**
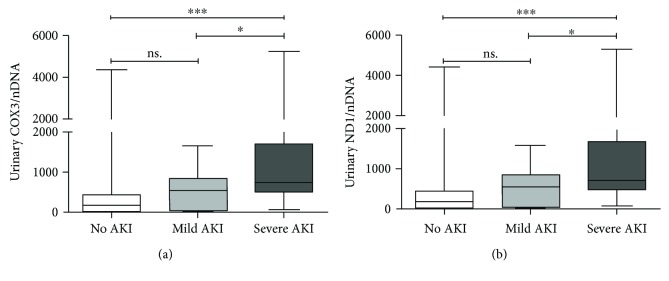
Urinary mitochondrial DNA (UmtDNA) copy number is associated with severity of renal injury in patients with diagnosis of sepsis. Patients were stratified into three groups: no AKI (*n* = 53), mild AKI (*n* = 18), and severe AKI (*n* = 34). Urinary COX3/nDNA (a) and ND1/nDNA (b) were significantly increased in severe AKI compared with those in no AKI or mild AKI. ^∗∗∗^ *P* < 0.001; ^∗^ *P* < 0.05. NS: no significance.

To evaluate whether UmtDNA can reflect renal dysfunction in humans, the level of UmtDNA in urine was detected by quantitative PCR. The UmtDNA values in AKI patients were compared with those in patients without AKI. The results showed that urinary mtDNA/nDNA was significantly elevated in septic AKI group (COX3: median = 672.1, IQR 389.9–1174.3; ND1: median = 692.1, IQR 445.2–1232.5) versus that in sepsis patients without AKI (COX3: median = 177.6, IQR 37.0–429.8; ND1: median = 178.6, IQR 39.9–441.4) (Supplementary Figures 2A-2B). To further investigate the effectiveness of UmtDNA in predicting the severity of AKI, enrolled patients were divided into three groups: no AKI (*n* = 53), mild AKI (*n* = 18), and severe AKI (*n* = 34). The UmtDNA level in the mild AKI patients was higher than that in the no AKI group; however, the difference did not reach statistical significance. Additionally, the UmtDNA level was significantly elevated in severe AKI (COX3: median = 749.6, IQR 489.2–1711.1; ND1: median = 766.7, IQR 479.2–1678.6) versus that in no AKI (COX3: median = 177.6, IQR 37.0–429.8; ND1: median = 178.6, IQR 39.9–441.4) or mild AKI (COX3: median = 550.2, IQR 72.3–855.9; ND1: median = 550.4, IQR 71.3–861.6) (Figure 2).

**Figure 3:**
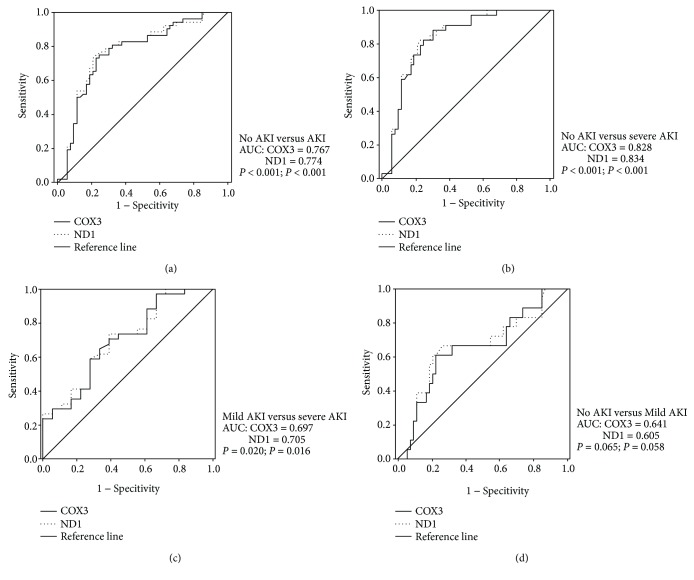
The UmtDNA copy number predicts the occurrence and progression of AKI in septic patients. (a–d) Area under the receiver operating characteristic curve (AUC) analysis was performed comparing different degrees of renal dysfunction following sepsis.

ROC curve analysis was performed to evaluate the diagnostic effectiveness for predicting AKI progression. The urine COX3/nDNA and ND1/nDNA ratios predicted the occurrence of AKI with an area under the curve (AUC) of 0.767 and 0.774, corresponding to a sensitivity and specificity of 73.1% and 77.4% and a sensitivity and specificity of 78.8% and 71.7%, respectively (Figure 3(a)). The diagnostic efficacy was elevated as a predictor of severe AKI with no AKI (COX3: AUC = 0.828, *P* < 0.001; ND1: AUC = 0.834, *P* < 0.001) and mild AKI patients (COX3: AUC = 0.697, *P* = 0.020; ND1: AUC = 0.705, *P* = 0.016) (Figures 3(b) and 3(c)). However, no significance was found between the no AKI and mild AKI groups (COX3: AUC = 0.641, *P* = 0.065; ND1: AUC = 0.605, *P* = 0.058) (Figure 3(d)).

### 3.4. UmtDNA Correlates with Marker of Renal Injury Following Sepsis

**Figure 4:**
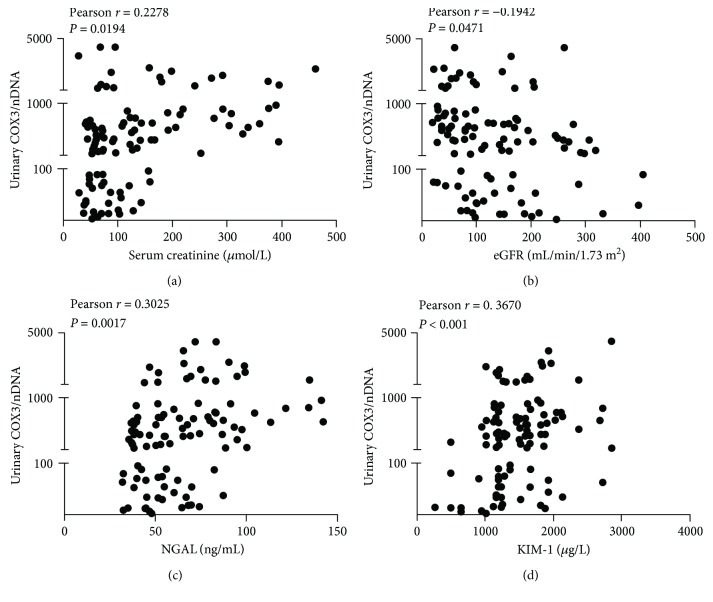
Urinary mitochondrial DNA copy number correlates with markers of renal injury and dysfunction. Urinary COX3/nDNA levels negatively correlated with estimated glomerular filtration rate (eGFR; b) but positively with serum creatinine (a), urinary neutrophil gelatinase-associated lipocalin (NGAL; c), and kidney injury molecule-1 (KIM-1; d) levels in septic patients.

To assess the ability of UmtDNA for the judgement of kidney dysfunction, we compared UmtDNA with other markers of renal dysfunction in the present study. Urinary NGAL and KIM-1 as new markers of renal damage and progression were similarly elevated in septic AKI compared with those in no AKI patients. NGAL, but not KIM-1, correlated positively with plasma creatinine levels in our study (Supplementary Figures 2C-2D). In patients with sepsis, urinary COX3/nDNA levels correlated inversely with eGFR and directly with plasma creatinine, urinary NGAL, and KIM-1 levels (Figures 4(a)–4(d)), and the correlation to urinary ND1/nDNA levels was not altered (Supplementary Figure 3). These correlations remained significant when AKI and no AKI were each considered alone (all *P* < 0.05).

### 3.5. UmtDNA Is Increased in Rats after Sepsis-Associated AKI and Correlates with Renal Dysfunction

**Figure 5:**
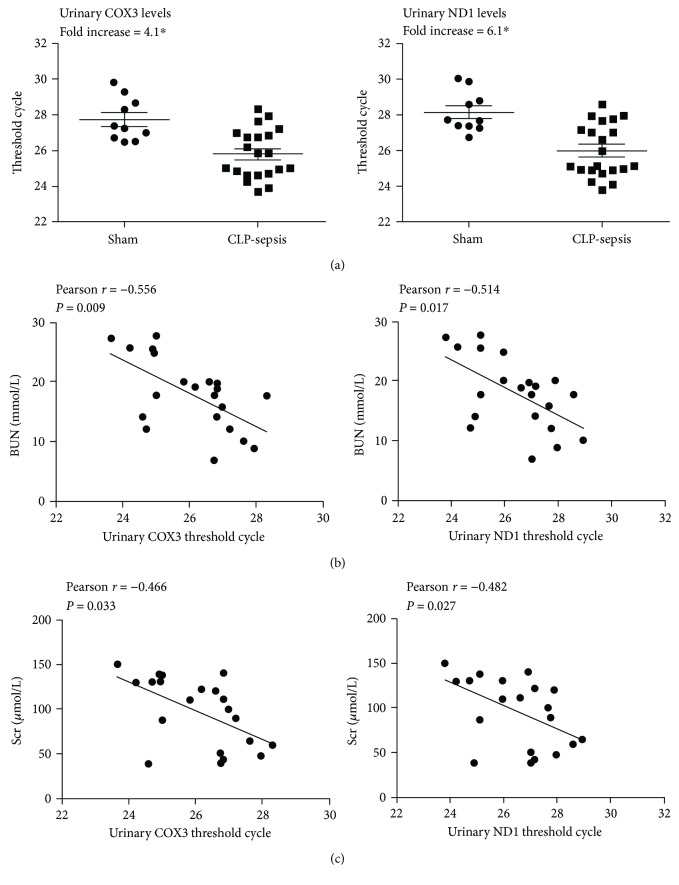
Urinary mitochondrial DNA (UmtDNA) levels are increased and correlated with renal injury following cecal ligation and puncture (CLP). Rats underwent sham surgery and CLP, and rats were placed in metabolic cages after CLP for urine collection. UmtDNA levels were significantly increased following sepsis induced by CLP (a) and correlated with serum creatinine (b) and BUN (c). ^∗^ *P* < 0.05.

To investigate the role of UmtDNA in the kidney, a rat model of sepsis-induced AKI by CLP was used. The rats were divided into sham surgery (*n* = 10) and CLP (*n* = 28) for 24 h. All rats produced adequate urine for detection of UmtDNA, and 7 rats died within 24 h postsurgery and were thus excluded from further analyses. BUN and plasma creatinine were significantly elevated at 24 h after CLP surgery (Supplementary Figure 4). UmtDNA could not be normalized to nDNA because of undetectable contents of GAPDH in rat urine. Therefore, UmtDNA levels were quantified as qPCR Tc, with each cycle doubling the DNA quantity until a detection threshold was achieved, and higher Tc represented lower levels of UmtDNA DAMPs. UmtDNA levels in the sepsis group showed a significant increase versus those in the sham group (Figure 5(a)). Moreover, UmtDNA significantly correlated with plasma creatinine (COX3: *r* = −0.466, *P* = 0.033; ND1: *r* = −0.482, *P* = 0.027) and blood urea nitrogen (COX3: *r* = −0.556, *P* = 0.009; ND1: *r* = −0.514, *P* = 0.017) (Figures 5(b) and 5(c)).

### 3.6. UmtDNA Predicts the Occurrence of AKI Development in Rats Following CLP

**Figure 6:**
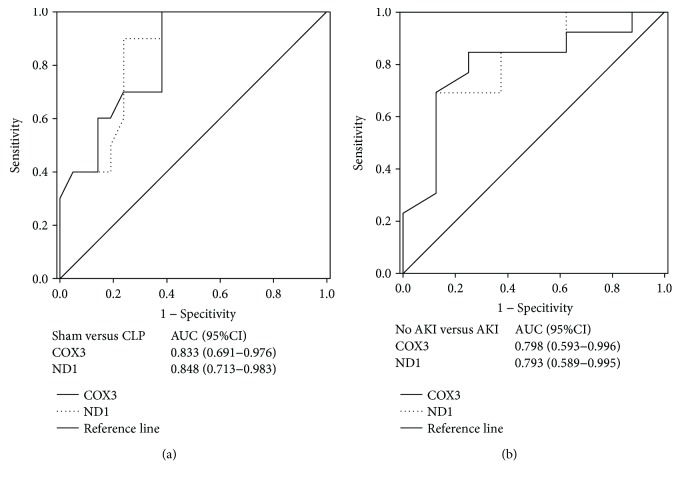
Urinary mitochondrial DNA (UmtDNA) is a biomarker of renal dysfunction following cecal ligation and puncture- (CLP) induced AKI in rats. Receiver operating characteristic curves were constructed for UmtDNA comparing (a) the sham group to rats that underwent CLP or (b) no AKI rats to AKI rat following sepsis. AKI: acute kidney injury.

Analysis of the ROC curve was performed to evaluate the capacity of UmtDNA to predict the occurrence of AKI in rats following CLP-induced sepsis. The UmtDNA COX3 and ND1 Tc number significantly predicted AKI development in rats undergoing CLP versus sham control rats, within an AUC of 0.833 and 0.848, corresponding to a sensitivity and specificity of 90.0% and 61.9% and a sensitivity and specificity of 90.0% and 76.2%, respectively (Figure 6(a)). There were several rats with no increase in BUN in the sepsis group. As previously defined, the AKI and no AKI group were classified according to an increase of >2 SD exceed a historical sham BUN level [14]. The cutoff of BUN in our study was ≥17.4 mmol/L, and rats below this BUN cutoff were excluded from the analysis. Using ROC curve analysis, UmtDNA COX3 and ND1 levels were significantly predictive of AKI development versus no AKI after CLP-induced sepsis with AUC of 0.798 and 0.793, corresponding to a sensitivity and specificity of 75.0% and 84.6% and a sensitivity and specificity of 87.5% and 69.2%, respectively (Figure 6(b)).

### 3.7. Renal mtDNA Copy Number and Mitochondrial Gene Expression Are Negatively Associated with UmtDNA in Sepsis-Associated AKI

**Figure 7:**
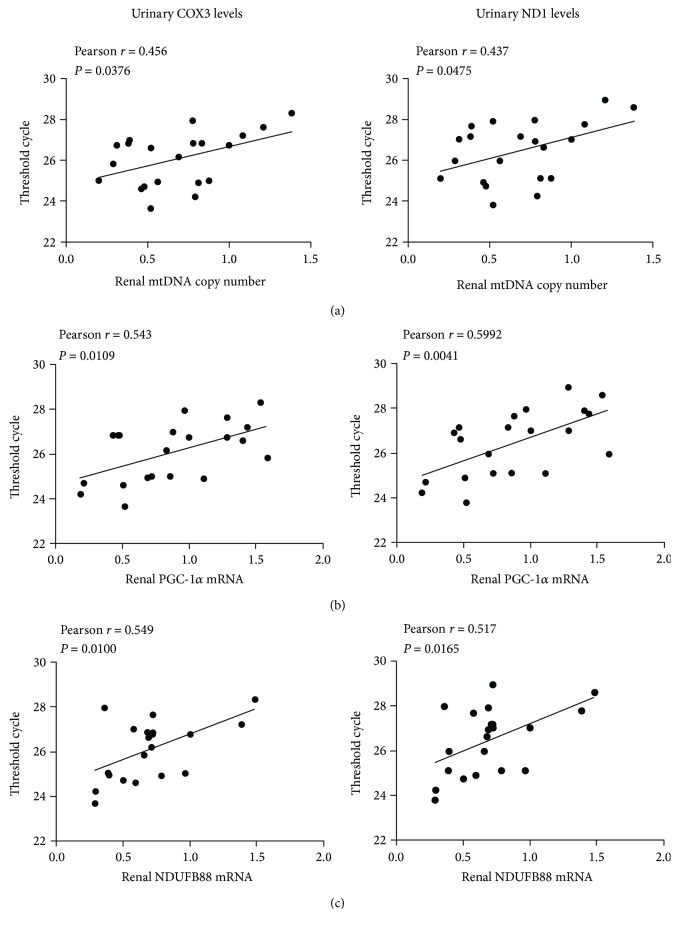
Urinary mitochondrial DNA (UmtDNA) is associated with depletion of the renal mtDNA copy number and mitochondrial gene expression. UmtDNA correlates with the relative (a) renal mtDNA copy number, (b) PGC-1*α* and (c) NDUFB8.

The renal cortical mtDNA copy number and transcriptional expression of PGC-1*α* and NDUFB8 were evaluated using qPCR to assess the integrity of renal mitochondria. The cortical mtDNA copy number in the kidney was reduced following sepsis, and inversely correlated with urinary COX3 (*r* = 0.456, *P* = 0.0376) and ND1 (*r* = 0.437, *P* = 0.0475) levels. Similarly, mRNA expression of PGC-1*α* (COX3: *r* = 0.543, *P* = 0.0109; ND1: *r* = 0.5992, *P* = 0.0041), and NDUFB8 (COX3: *r* = 0.549, *P* = 0.0100; ND1: *r* = 0.517, *P* = 0.0165) was reduced after sepsis-induced AKI, and their expression was also inversely associated with UmtDNA levels (Figure 7).

### 3.8. UmtDNA Is Correlated with the Decrease in Renal ATP Levels and Renal Tubular Injury

**Figure 8:**
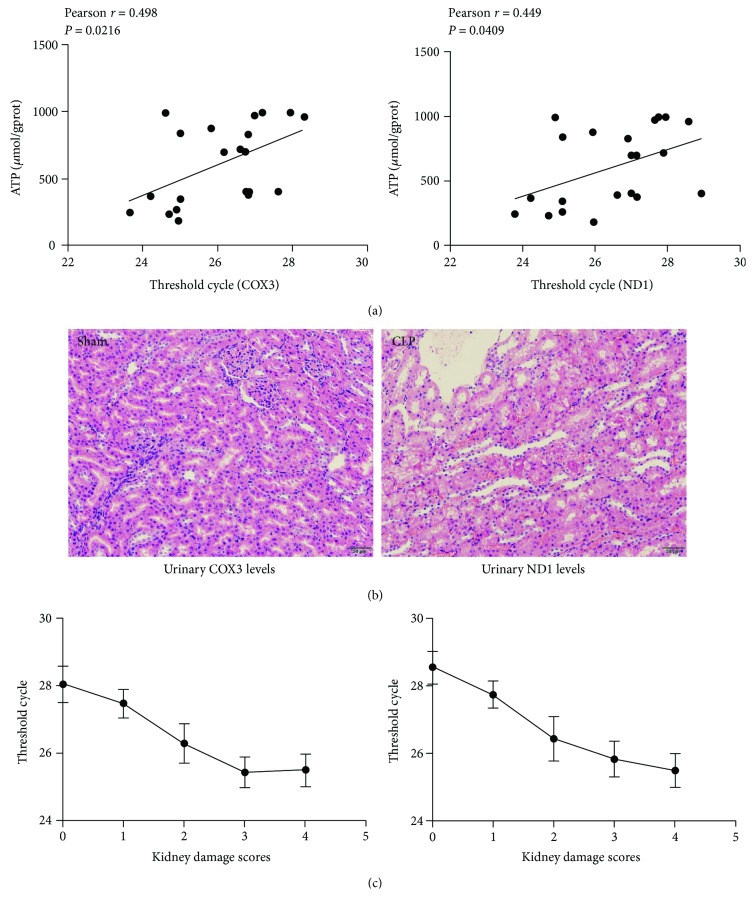
Urinary mitochondrial DNA (UmtDNA) is associated with the reduction of (a) renal ATP levels and (b) renal tubular injury scores (hematoxylin and eosin staining; original magnification ×200; scale bars represent 50 *μ*m).

The renal tubule is one of the richest tissues in terms of number and density of mitochondria. Mitochondrial ATP levels, an indicator of mitochondrial activity, were quantified to estimate the renal tubule dysfunction.  Figure 8(a) showed that ATP concentrations were markedly reduced in the I/R mice and were inversely correlated with UmtDNA levels (COX3: *r* = 0.498, *P* = 0.0216; ND1: *r* = 0.449, *P* = 0.0409). Moreover, the kidney of rats in the CLP-operated group showed obvious edema of the renal proximal tubular epithelial cells, narrowing of the renal proximal tubular lumina, and brush border loss as well as sporadic inflammatory cell infiltration (Figure 8(b)). Interestingly, Figure 8(c) showed that there were intriguing trends relating renal tubular injury scores and UmtDNA levels in the present study.

## 4. Discussion

The current study demonstrates that UmtDNA is elevated in septic patients with AKI and correlates with other markers of renal dysfunction, suggesting mitochondrial damage in the kidney in sepsis-induced AKI patients. Furthermore, correlations of UmtDNA with mitochondrial gene expression in rat models of sepsis-associated AKI indicate that the predictive efficacy of UmtDNA for AKI severity may arise from its capacity to predict the degree of renal mitochondrial injury and dysfunction, which can prevent the renal repair process.

**Figure 9:**
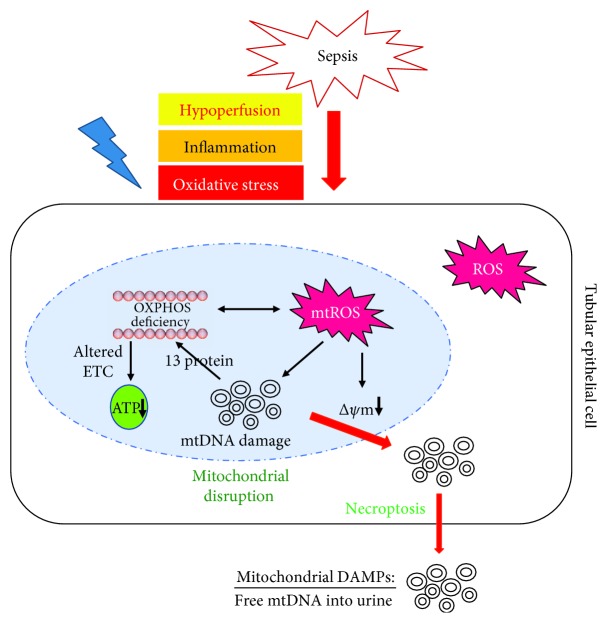
Mitochondrial DNA as a damage-associated molecular pattern. During sepsis (hypoperfusion/inflammation/oxidative stress), the mitochondria causes mitochondrial membrane potential transition and reactive oxygen species generation, which lead to the release of damaged mtDNA into the cytosol. Cytosolic mtDNA can also be secreted into the extracellular space (urine) by necroptosis-mediated programed cell death in renal tubular epithelial cells. ETC: electron transport chain; mtROS: mitochondrial ROS; mtDNA: mitochondrial DNA.

Emerging evidence demonstrates a disruption of mitochondrial integrity in the pathogenesis of sepsis-associated AKI. Sepsis activates several pathological mechanisms linked to mitochondria, including hypoperfusion, oxidative stress, and the inflammatory response. Activation of these pathways could produce a large amount of reactive oxygen species (ROS) and decrease antioxidant defenses, thus disrupting mitochondrial integrity (Figure 9). Ultrastructural changes in the mitochondria are observed in the kidney tubular cells during sepsis-induced AKI. These changes include mitochondrial impairment, swelling, cellular death, and finally the release of mitochondrial contents into the extracellular space, leading to a vicious cycle of renal damage, which may represent different levels of damage to mitochondria [4]. Additionally, high energies are needed in the repair of the renal tubular epithelium, and thus renal structural and functional recovery depend on mitochondrial function [14]. Because of the vital roles of mitochondria in renal recovery, renal mitochondrial fragments can be a reliable biomarker of AKI progression. In contrast, a lack of clinical methods to detect mitochondrial function has restricted investigations of the relationship between mitochondrial disruption and renal dysfunction in a human study [17–19].

MtDNA is vulnerable to damage targeted by ROS because it has no effective repair mechanisms, and increased mitochondrial ROS generation can decrease mitochondrial membrane potential, leading to impairment of membrane integrity [20, 21]. These changes could subsequently permit leakage of mtDNA into the cytosol. Furthermore, one of the proposed mechanisms by which mtDNA is translocated to the extracellular space is via necroptosis [21–23]. Therefore, disruption of mitochondrial integrity in the renal tubular epithelial cells can cause the release of mitochondrial DAMPs into the urine, where they could be considered as surrogate biomarkers of renal mitochondrial damage and are responsible for the progression of renal injury (Figure 9). Urinary COX3 and ND1 mtDNA levels were compared between no AKI patients and AKI patients following sepsis, and their relationships with markers of renal injury were also investigated. We found that UmtDNA was dramatically elevated in severe AKI following sepsis compared with that in no AKI or mild AKI, and the UmtDNA level was associated with an increased risk of AKI development and progression, suggesting mitochondrial injury and impaired energy production in sepsis-induced AKI.

Importantly, the UmtDNA copy number was positively related to urinary levels of tubular injury markers. NGAL, presumably from distal tubular origins at least in experimental AKI, is the most frequently described human AKI biomarker, and KIM-1 as a proximal tubular injury biomarker has been validated to be an effective marker for the early detection of AKI. The present study also showed that urinary NGAL and KIM-1 in sepsis patients correlated negatively with renal function but positively with UmtDNA levels, suggesting renal mitochondrial stress and injury. Expectedly, UmtDNA levels correlated directly with plasma creatinine and inversely with eGFR, which were consistent with our previous study in critically ill patients. However, Whitaker et al. [14] demonstrated no correlations between UmtDNA and plasma creatinine in patients following cardiopulmonary bypass surgery. We argue that the differences are due to diverse patient cohorts, a heterogeneous surgical intervention, and the poor sensitivity of plasma creatinine. Furthermore, the small sample size in our study could also have tempered these conclusions. Thus, larger study in septic patients is needed to verify the prognostic effectiveness of UmtDNA for AKI progression.

Current diagnosis and staging of AKI depend on changes in traditional markers of renal injury, like serum creatinine and urine output [24], but the utility of these markers for predicting AKI progression in sepsis is limited. Thus, more specific and sensitive biomarkers are being developed to enhance the predictive power. The diagnostic value of newer markers, including NGAL and KIM-1, is satisfactory. Urinary NGAL and KIM-1 have shown promising results for predicting patient mortality and AKI development in sepsis, reflecting kidney inflammation. Parr et al. investigated the capacity of a series of renal markers, such as urinary NGAL, KIM-1, L-FABP, and interleukin-18, to predict AKI development in critically ill patients using the KDIGO criteria [25]. Urinary L-FABP was demonstrated to be the best biomarker in predicting AKI progression with an AUC of 0.79. In the present study, the predictive power of UmtDNA levels was compared favorably (COX3: AUC = 0.767; ND1: AUC = 0.774, no AKI versus AKI). Similarly, our study also showed that the diagnostic effectiveness of UmtDNA (COX3: AUC = 0.767; ND1: AUC = 0.774) better distinguished no AKI and AKI compared with the NGAL (AUC = 0.751) and KIM-1(AUC = 0.694) (Supplementary Figure 5).

Due to unavailable renal samples from septic patients, a rat model of CLP-induced sepsis was used to investigate the physiological association between UmtDNA levels and mitochondrial integrity in the renal cortex. We showed that mitochondrial integrity was persistently damaged after sepsis-associated AKI in rats. Funk and Schnellmann [19] suggested that persistent mitochondrial damage was related to a failure of the renal repair process. In the present study, the UmtDNA was significantly increased in sepsis and sepsis-associated AKI compared with that in the sham group and correlated with renal function assessed by plasma creatinine and BUN. These data differed from the results of Whitaker et al., as no correlation was observed between UmtDNA and BUN. This difference is likely due to the specific animal model and different sensitivity and performance of BUN in various diseases. Overall, these data demonstrate that rat CLP provides a reliable model of the UmtDNA response observed in sepsis and a platform for the detection of mitochondrial indexes in the kidney.

Structural and functional renal recovery depends on persistent activation of tissue repair processes, especially for the renal tubule [26]. The renal cortical ATP level is an important determinant of energy metabolism and is associated with mitochondrial function and dynamics [27]. Energy is necessarily required to repair the renal tubular epithelium, and thus mitochondrial function is critical for renal recovery [14]. Importantly, a recent animal study has shown that mitochondria-targeted therapy significantly protects against sepsis-induced renal alternations by modulating tubular cell apoptosis and decreasing ROS-related mitochondrial damage [5]. Similarly, Dare et al. also revealed that improving mitochondrial homeostasis and function by injection of MitoQ (mitochondria-targeted antioxidant) has the potential to restore renal dysfunction due to ischemia reperfusion injury [28]. However, clinically useful and noninvasive assessments of renal repair process by mitochondria-targeted therapy are limited for patients. Our study demonstrated that UmtDNA correlated with renal cortical ATP levels and was associated with renal damage graded by the tubular injury score, which indicated that UmtDNA levels can be used as a potent marker of the potential of renal repair and a prognostic index of functional recovery in the kidney.

The MtDNA copy number and mRNA expression of PGC-1*α* and NDUFB8 from the renal cortex were investigated and showed significant decreases following sepsis-induced AKI in the present study. The correlation of UmtDNA levels with the mtDNA copy number and mitochondrial gene expression indicated that UmtDNA reflected kidney homeostasis during sepsis. Based on the above results, we suggested that the predictive ability of UmtDNA for AKI severity was due to its capacity to predict the degree of mitochondrial dysfunction and fission, which could lead to reduction of ATP production, cellular functions, and structural alterations, as well as inhibition of renal repair processes. Recent animal studies have clearly suggested that the disruption of mitochondrial homeostasis in the early stages of acute kidney injury is a major factor driving tubular injury and inhibiting renal recovery [29]. However, negative correlations of mitochondrial gene expression to elevated UmtDNA do not casually link mitochondrial homeostasis to structural and functional recovery from AKI. Therefore, future studies are needed to explore how the modulation of UmtDNA level is associated with renal tissue repair and recovery following sepsis-induced AKI.

In addition to serving as a clinical biomarker of mitochondrial stress and dysfunction, circulating mtDNA has been shown to induce the inflammatory response following mitochondrial damage. We have already shown that circulating mtDNA is linked to the occurrence of multiple organ dysfunction syndrome and mortality [15]. Importantly, mtDNA contributes to the activation of Toll-like receptor 9 and inflammasomes, propagating damage to distant organs following sepsis [21]. We have previously demonstrated that critically ill patients with sepsis display increased levels of plasma mtDNA compared with those without sepsis [15]. Nevertheless, no differences were observed between AKI and no AKI patients following sepsis in the present study. Furthermore, circulating mtDNA did not correlate with the biomarkers of systemic inflammation and renal dysfunction. He et al. [30] demonstrated that injection of mtDNA or mitochondrial debris failed to induce proteinuria and kidney injury in rodents, suggesting that circulating mtDNA may not be responsible for the renal function and dysfunction in critically ill patients. Similarly, a recent study showed that increased circulating mtDNA levels in systemic inflammatory response syndrome patients did not correlate with circulating mtDNA and renal disease severity, which indicated that AKI did not have an additional effect on circulating levels. The authors suggested that systemic mtDNA was probably not important in the vicious circle between systemic inflammatory response syndrome (SIRS) and AKI, whereas the correlation between UmtDNA and increased markers of renal inflammation indicated that renal mtDNA accumulation may be related to intrarenal inflammation and renal dysfunction in the pathophysiology of SIRS [31]. These data suggest that elevated circulating mtDNA due to sepsis does not lead to secondary AKI, whereas UmtDNA accumulation correlates with renal mitochondrial disruption and sepsis-induced renal dysfunction. Furthermore, compared with plasma mtDNA and other biomarkers of renal dysfunction, assessment of UmtDNA is noninvasive and easily collected, which thus can be consecutively measured to evaluate changes in the renal function and the kidney repair process in human sepsis.

## 5. Conclusion

Our studies showed that UmtDNA is associated with mitochondrial disruption following sepsis-induced AKI. Furthermore, we provide preliminary evidence that UmtDNA may be a valuable assessment for investigations of mitochondrial dysfunction and injury in human sepsis-induced AKI and the development of mitochondria-targeted therapy for renal disease.

## Figures and Tables

**Table 1:** Demographic and clinical variables and outcomes of patients stratified by the presence of AKI.

Parameters	Sepsis patients enrolled in SICU			*P* value
	All (*n* = 105)	No AKI (*n* = 53)	AKI (*n* = 52)	
Demographic data				
Age, mean (SD), y	51.3 ± 14.6	51.2 ± 14.5	51.4 ± 14.9	0.946
Male, *n* (%)	82 (78.1%)	38 (71.7%)	44 (84.6%)	0.110
BMI, mean (SD)	21.2 ± 3.0	21.1 ± 2.8	21.2 ± 3.2	0.628
Primary disease, *n* (%)				0.649
Traffic accident	8 (7.6%)	3 (5.6%)	5 (9.6%)	
Injury^a^	13 (12.4%)	5 (9.4%)	8 (15.4%)	
Surgical complication^b^	78 (74.3%)	42 (79.3%)	36 (69.2%)	
Others	6 (5.7%)	3 (5.7%)	3 (5.8%)	
Comorbidity, *n* (%)				
Hypertension	19 (18.1%)	11 (20.8%)	8 (15.4%)	0.475
Diabetes mellitus	12 (11.4%)	5 (9.4%)	7 (13.5%)	0.517
Cardiovascular disease	5 (4.8%)	3 (5.7%)	2 (3.8%)	0.663
Site of infections, *n* (%)				0.903
Abdominal	51 (48.6%)	27 (50.9%)	24 (46.2%)	
Pulmonary	25 (23.8%)	11 (20.8%)	14 (26.9%)	
Urinary	6 (5.7%)	3 (5.7%)	3 (5.8%)	
Others	23 (21.9%)	12 (22.6%)	11 (21.1%)	
ICU admission				
APACHE II score, mean (SD)	13.8 ± 5.3	12.2 ± 3.4	15.5 ± 6.4	0.001
SOFA score, mean (SD)	9.1 ± 3.8	8.1 ± 2.7	10.1 ± 4.5	0.006
Mechanical ventilation, *n* (%)	40 (38.1%)	15 (28.3%)	25 (48.1%)	0.037
Leukocyte count, mean (SD), ×109/L	14.7 ± 2.1	14.4 ± 1.8	15.1 ± 2.3	0.086
CRP, mean (SD), mg/L	72.1 ± 30.2	68.6 ± 25.2	75.6 ± 38.7	0.276
PCT, mean (SD), ng/mL	4.0 ± 1.7	3.7 ± 1.4	4.3 ± 1.6	0.043
IL-6, mean (SD), pg/mL	197.5 ± 85.4	177.2 ± 77.8	218.2 ± 95.3	0.017
Plasma sodium, mean (SD), mmol/L	140.5 ± 6.2	141.1 ± 6.9	139.8 ± 5.8	0.299
Plasma potassium, mean (SD), mmol/L	4.2 ± 1.1	4.3 ± 1.2	4.1 ± 0.9	0.336
Lactate, mean (SD), mmol/L	2.3 ± 1.5	2.1 ± 1.1	2.6 ± 1.9	0.104
eGRF, median (IQR)	59.6 [32.0–92.5]	73.3 [49.0–114.0]	42.4 [22.1–77.0]	<0.001
Plasma creatinine, median (IQR), *μ*mol/L	81.0 [116.0–177.0]	90.0 [70–129.0]	151.0 [96.0–287.0]	<0.001
Urinary NGAL, median (IQR), ng/mL	60.3 [44.6–82.1]	50.3 [39.4–66.2]	74.0 [52.3–92.9]	<0.001
Urinary KIM-1, median (IQR), ng/mL	1642.0 [1198.5–1819.7]	1236.1 [1060.8–1604.6]	1615.3 [1241.3–1294.5]	0.001
Outcomes				
Septic shock	18 (17.1%)	5 (9.4%)	13 (25%)	0.34
Receiving RRT, *n* (%)	19 (18.1%)	0(%)	19 (28.8%)	<0.001
Hospital LOS, mean (SD), d	23.7 ± 8.7	21.2 ± 6.7	26.1 ± 9.8	0.003
Hospital mortality, *n* (%)	18 (17.1%)	5 (9.4%)	13 (25%)	0.34

BMI: body mass index; APACHE: Acute Physiology and Chronic Health Evaluation; SOFA: Sepsis-related Organ Failure Assessment; CRP: C-reaction protein; PCT: procalcitonin; NGAL: neutrophil gelatinase-associated lipocalin; KIM-1: kidney injury molecule-1; eGFR: estimated glomerular filtration; LOS: length of stay. ^a^Injury includes gunshot, falling, cuts, and bruising; ^b^Patients who developed into intra-abdominal infection after elective surgery were categorized as having surgical complication.

**Table 2:** Correlations between plasma mtDNA and markers of systemic inflammation and kidney injury in sepsis patients.

	Plasma COX3 levels	Plasma ND1 levels
*Inflammation*		
Leukocyte count	*r* = 0.10; *P* = 0.29	*r* = 0.13; *P* = 0.24
Plasma CRP	*r* = 0.034; *P* = 0.733	*r* = −0.146; *P* = 137
Plasma PCT	*r* = 0.14; *P* = 0.19	*r* = 0.19; *P* = 0.058
Plasma IL-6 (pg/mL)	*r* = 0.20; *P* = 0.04^∗^	*r* = 0.18; *P* = 0.08
*Renal Function*		
Plasma creatinine (*μ*mol/L)	*r* = 0.15; *P* = 0.11	*r* = 0.12; *P* = 0.14
Urinary NGAL	*r* = 0.11; *P* = 0.31	*r* = 0.09; *P* = 0.45
Urinary KIM-1	*r* = 0.08; *P* = 0.41	*r* = 0.05; *P* = 0.57

Linear regression was performed and Spearman's rank-order coefficients were calculated. CRP: C-reactive protein; PCT: procalcitonin; NGAL: neutrophil gelatinase-associated lipocalin; KIM-1: kidney injury molecule-1. ^∗^ *P* < 0.05 indicates significant correlations.

## Supplementary Materials

Supplementary Materials Supplementary Table 1: primers for qPCR analyses of the relevant sequences. Supplementary Figure 1: there is no significant difference in plasma mtDNA levels between AKI group and no AKI group. Supplementary Figure 2: urinary mtDNA was significantly elevated in septic AKI group versus that in sepsis patients without AKI (A-B); NGAL (C), but not KIM-1 (D), was correlated positively with plasma creatinine levels. ^∗∗∗^ *P* < 0.001. Supplementary Figure 3: urinary ND1/nDNA levels were correlated inversely with eGFR and directly with plasma creatinine, urinary NGAL, and KIM-1 levels. Supplementary Figure 4: BUN and plasma creatinine were significantly elevated at 24 h after cecal ligation and puncture surgery. ^∗^ *P* < 0.05, ^∗∗^ *P* < 0.01. Supplementary Figure 5: diagnostic effectiveness of urinary NGAL-1 and KIM-1 on the occurrence of AKI by ROC curve analysis.

## References

[1] Dai X., Zeng Z., Fu C., Zhang S., Cai Y., Chen Z. (2015). Diagnostic value of neutrophil gelatinase-associated lipocalin, cystatin C, and soluble triggering receptor expressed on myeloid cells-1 in critically ill patients with sepsis-associated acute kidney injury. *Critical Care*.

[2] Bellomo R., Kellum J. A., Ronco C. (2017). Acute kidney injury in sepsis. *Intensive Care Medicine*.

[3] Honore P. M., Nguyen H. B., Gong M. (2016). Urinary tissue inhibitor of metalloproteinase-2 and insulin-like growth factor-binding protein 7 for risk stratification of acute kidney injury in patients with sepsis. *Critical Care Medicine*.

[4] Parikh S. M., Yang Y., He L., Tang C., Zhan M., Dong Z. (2015). Mitochondrial function and disturbances in the septic kidney. *Seminars in Nephrology*.

[5] Zhao W. Y., Zhang L., Sui M. X., Zhu Y. H., Zeng L. (2016). Protective effects of sirtuin 3 in a murine model of sepsis-induced acute kidney injury. *Scientific Reports*.

[6] Karlsson M., Hara N., Morata S. (2016). Diverse and tissue-specific mitochondrial respiratory response in a mouse model of sepsis-induced multiple organ failure. *Shock*.

[7] Hinkelbein J., Böhm L., Braunecker S., Adler C., De Robertis E., Cirillo F. (2017). Decreased tissue COX5B expression and mitochondrial dysfunction during sepsis-induced kidney injury in rats. *Oxidative Medicine and Cellular Longevity*.

[8] Gao Y., Zeng Z., Li T. (2015). Polydatin inhibits mitochondrial dysfunction in the renal tubular epithelial cells of a rat model of sepsis-induced acute kidney injury. *Anesthesia & Analgesia*.

[9] Xu S., Gao Y., Zhang Q. (2016). SIRT1/3 activation by resveratrol attenuates acute kidney injury in a septic rat model. *Oxidative Medicine and Cellular Longevity*.

[10] Eirin A., Saad A., Tang H. (2016). Urinary mitochondrial DNA copy number identifies chronic renal injury in hypertensive patients. *Hypertension*.

[11] Eirin A., Saad A., Woollard J. R. (2017). Glomerular hyperfiltration in obese African American hypertensive patients is associated with elevated urinary mitochondrial-DNA copy number. *American Journal of Hypertension*.

[12] Hu Q., Ren J., Wu J. (2017). Urinary mitochondrial DNA levels identify acute kidney injury in surgical critical illness patients. *Shock*.

[13] Bellomo R., Ronco C., Kellum J. A., Mehta R. L., Palevsky P., Acute Dialysis Quality Initiative workgroup (2004). Acute renal failure – definition, outcome measures, animal models, fluid therapy and information technology needs: the Second International Consensus Conference of the Acute Dialysis Quality Initiative (ADQI) group. *Critical Care*.

[14] Whitaker R. M., Stallons L. J., Kneff J. E. (2015). Urinary mitochondrial DNA is a biomarker of mitochondrial disruption and renal dysfunction in acute kidney injury. *Kidney International*.

[15] Hu Q., Ren J., Wu J. (2017). Elevated levels of plasma mitochondrial DNA are associated with clinical outcome in intra-abdominal infections caused by severe trauma. *Surgical Infections*.

[16] Zafrani L., Ergin B., Kapucu A., Ince C. (2016). Blood transfusion improves renal oxygenation and renal function in sepsis-induced acute kidney injury in rats. *Critical Care*.

[17] Jesinkey S. R., Funk J. A., Stallons L. J. (2014). Formoterol restores mitochondrial and renal function after ischemia–reperfusion injury. *Journal of the American Society of Nephrology*.

[18] Stallons L. J., Funk J. A., Schnellmann R. G. (2013). Mitochondrial homeostasis in acute organ failure. *Current Pathobiology Reports*.

[19] Funk J. A., Schnellmann R. G. (2012). Persistent disruption of mitochondrial homeostasis after acute kidney injury. *American Journal of Physiology-Renal Physiology*.

[20] Nakahira K., Haspel J. A., Rathinam V. A. K. (2011). Autophagy proteins regulate innate immune responses by inhibiting the release of mitochondrial DNA mediated by the NALP3 inflammasome. *Nature Immunology*.

[21] Harrington J. S., Choi A. M. K., Nakahira K. (2017). Mitochondrial DNA in sepsis. *Current Opinion in Critical Care*.

[22] Kaczmarek A., Vandenabeele P., Krysko D. V. (2013). Necroptosis: the release of damage-associated molecular patterns and its physiological relevance. *Immunity*.

[23] Linkermann A., Green D. R. (2014). Necroptosis. *The New England Journal of Medicine*.

[24] Nisula S., Yang R., Poukkanen M. (2015). Predictive value of urine interleukin-18 in the evolution and outcome of acute kidney injury in critically ill adult patients. *British Journal of Anaesthesia*.

[25] Parr S. K., Clark A. J., Bian A. (2015). Urinary L-FABP predicts poor outcomes in critically ill patients with early acute kidney injury. *Kidney International*.

[26] Bonventre J. V., Yang L. (2011). Cellular pathophysiology of ischemic acute kidney injury. *The Journal of Clinical Investigation*.

[27] Bhargava P., Schnellmann R. G. (2017). Mitochondrial energetics in the kidney. *Nature Reviews Nephrology*.

[28] Dare A. J., Bolton E. A., Pettigrew G. J., Bradley J. A., Saeb-Parsy K., Murphy M. P. (2015). Protection against renal ischemia–reperfusion injury *in vivo* by the mitochondria targeted antioxidant MitoQ. *Redox Biology*.

[29] Li Y., Ye Z., Lai W. (2017). Activation of sirtuin 3 by silybin attenuates mitochondrial dysfunction in cisplatin-induced acute kidney injury. *Frontiers in Pharmacology*.

[30] He J., Lu Y., Xia H. (2015). Circulating mitochondrial DAMPs are not effective inducers of proteinuria and kidney injury in rodents. *PLoS One*.

[31] Jansen M. P. B., Pulskens W. P., Butter L. M. (2017). Mitochondrial DNA is released in urine of SIRS patients with acute kidney injury and correlates with severity of renal dysfunction. *Shock*.

