# Supplementary material for: Urinary Mitochondrial DNA Identifies Renal Dysfunction and Mitochondrial Damage in Sepsis-Induced Acute Kidney Injury
Source: Oxid Med Cell Longev. 2018 Feb 26;2018:8074936. doi: 10.1155/2018/8074936 (PMC5846356; doi:10.1155/2018/8074936)
Supplement: Supplementary Materials — Supplementary Table 1: primers for qPCR analyses of the relevant sequences. Supplementary Figure 1: there is no significant difference in plasma mtDNA levels between AKI group and no AKI group. Supplementary Figure 2: urinary mtDNA was significantly elevated in septic AKI group versus that in sepsis patients without AKI (A-B); NGAL (C), but not KIM-1 (D), was correlated positively with plasma creatinine levels. ∗∗∗ P < 0.001. Supplementary Figure 3: urinary ND1/nDNA levels were correlated inversely with eGFR and directly with plasma creatinine, urinary NGAL, and KIM-1 levels. Supplementary Figure 4: BUN and plasma creatinine were significantly elevated at 24 h after cecal ligation and puncture surgery. ∗ P < 0.05, ∗∗ P < 0.01. Supplementary Figure 5: diagnostic effectiveness of urinary NGAL-1 and KIM-1 on the occurrence of AKI by ROC curve analysis. [file 8074936.f1.docx]

Supplementary Table 1. Primers for qPCR analyses of the relevant sequences.

|  | **DNA Copy Number Quantification/Detection** | |
| --- | --- | --- |
| **Homo sapiens** | Human *GAPDH* | F: CCTGAGGGTTCTTTGTGCTGA  R: AAAGGCTCAACCTTCCCCAT |
|  | Human *COX3* | F: AGGCATCACCCCGCTAAAT  R: GGTGAGCTCAGGTGATTGATACTC |
|  | Human *ND1* | F: AGTCACCCTAGCCATCATTCTACT  R: GGAGTAATCAGAGGTGTTCTTGTGT |
| **Rattus norvegicus** | Rat *GAPDH* | F: TGGTAACCAGGCGTCCGATA  R: GGTGCAGCGATGCTTTACTTTC |
|  | Rat *COX3* | F: CAGCCTAGTTCCTACCCACG  R: GGCTCATGTAATTGAGACTCCTG |
|  | Rat *ND1* | F: CTACGCAAAGGCCCCAACAT  R: TAGAGCTAGTGTAAGGGAGAGGG |
| **Gene Expression Quantification/Detection(mRNA)** | | |
| **Rattus norvegicus** | *PGC1α* | F: AGGAAATCCGAGCTGAGCTGAACA  R: GCAAGAAGGCGACACATCGAACAA |
|  | *NDUFB8* | F: GATGCTCCCTAACCGCTCAC  R: CATGAAAGCCACAAAGCCGA |
|  | *GAPDH* | F: ATGACTCTACCCACGGCAAG  R: GGAAGATGGTGATGGGTTTC |

For DNA copy number quantification, the two primers of patients’ and rats’ urinary mtDNA we use are listed in Supplementary Table 1, including the rats’ renal mRNA primers for gene expression detection.


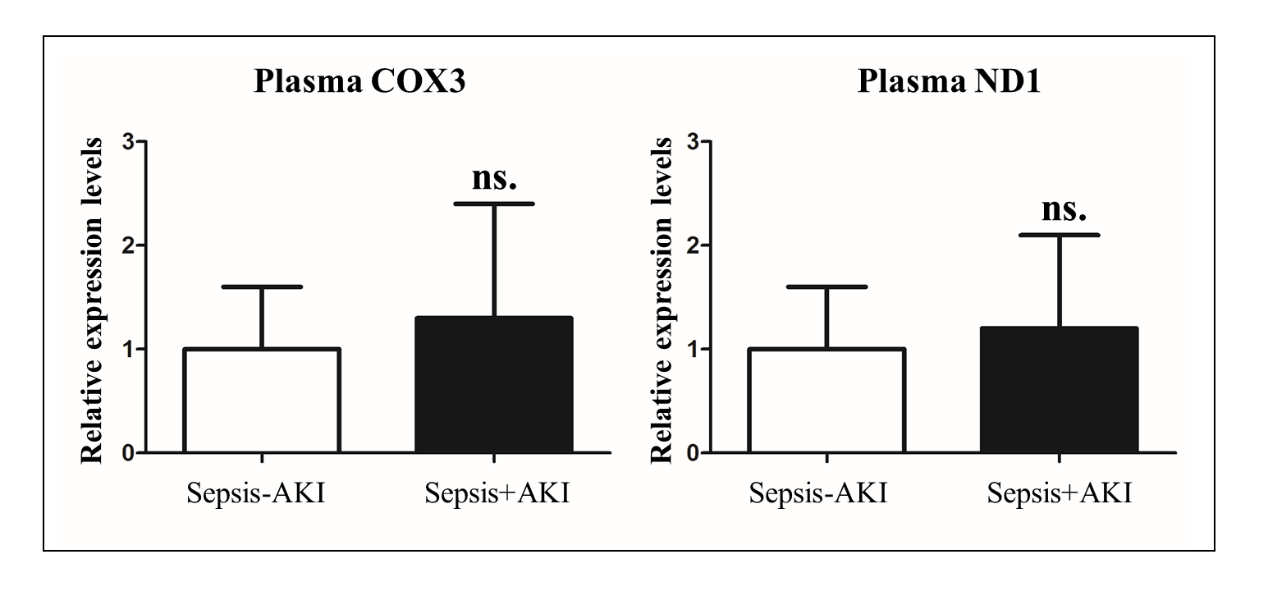


**Supplementary Figure 1.** There is no significant difference in plasma mtDNA levels between AKI group and no AKI group.


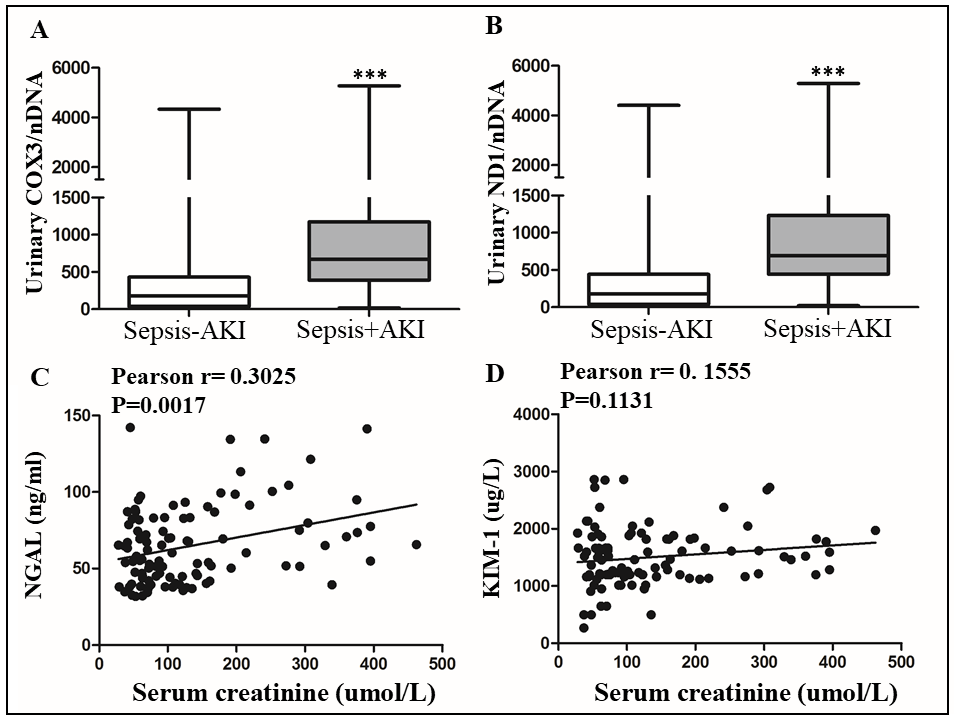


**Supplementary Figure 2.** Urinary mtDNA was significantly elevated in septic AKI group versus those sepsis patients without AKI (**A-B**); NGAL (**C**), but not KIM-1 (**D**), was correlated positively with plasma creatinine levels. ***P<0.001.


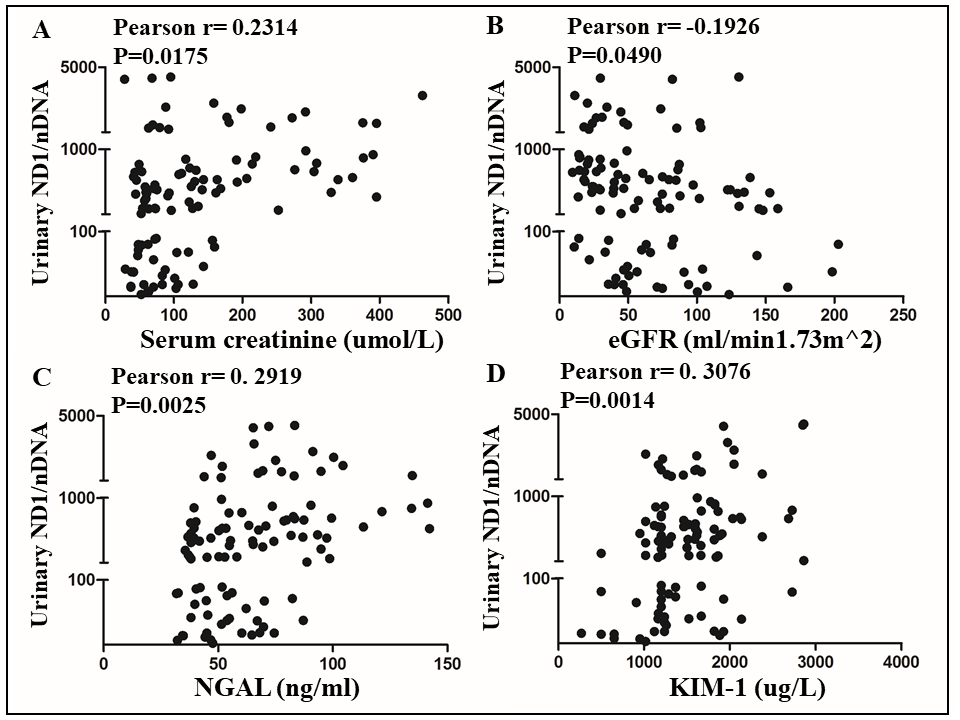


**Supplementary Figure 3.** Urinary ND1/nDNA levels was correlated inversely with eGFR, and directly with plasma creatinine, urinary NGAL and KIM-1 levels.


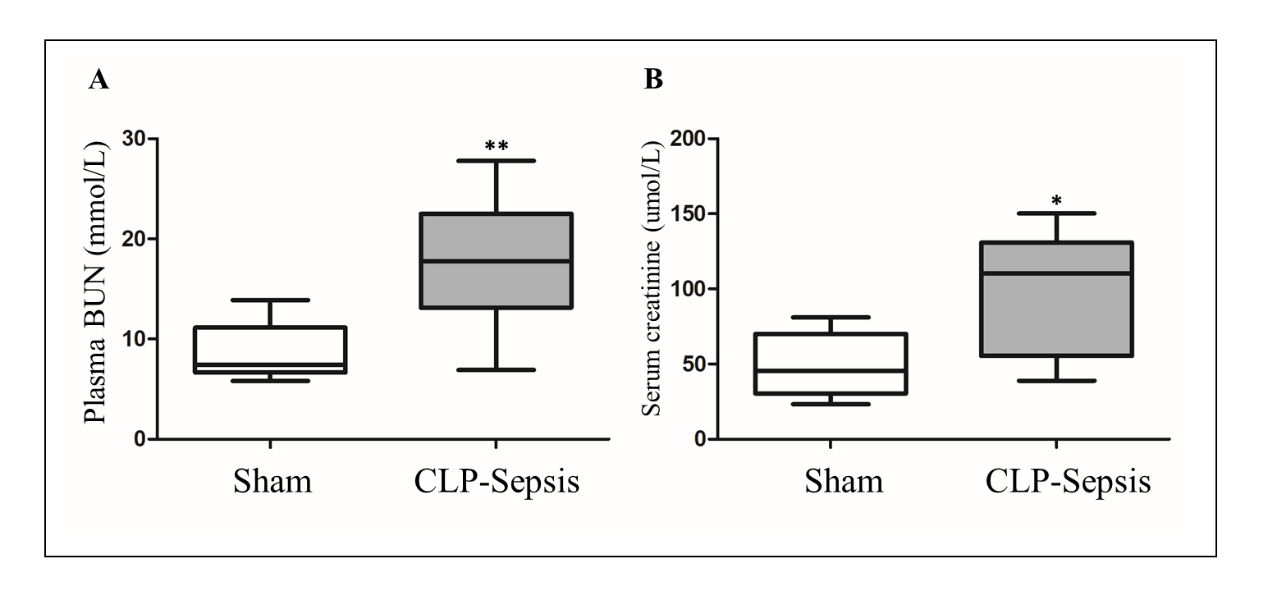


**Supplementary Figure 4.** BUN and plasma creatinine were significantly elevated at 24h after cecal ligation and puncture surgery. *P<0.05, **P<0.01.


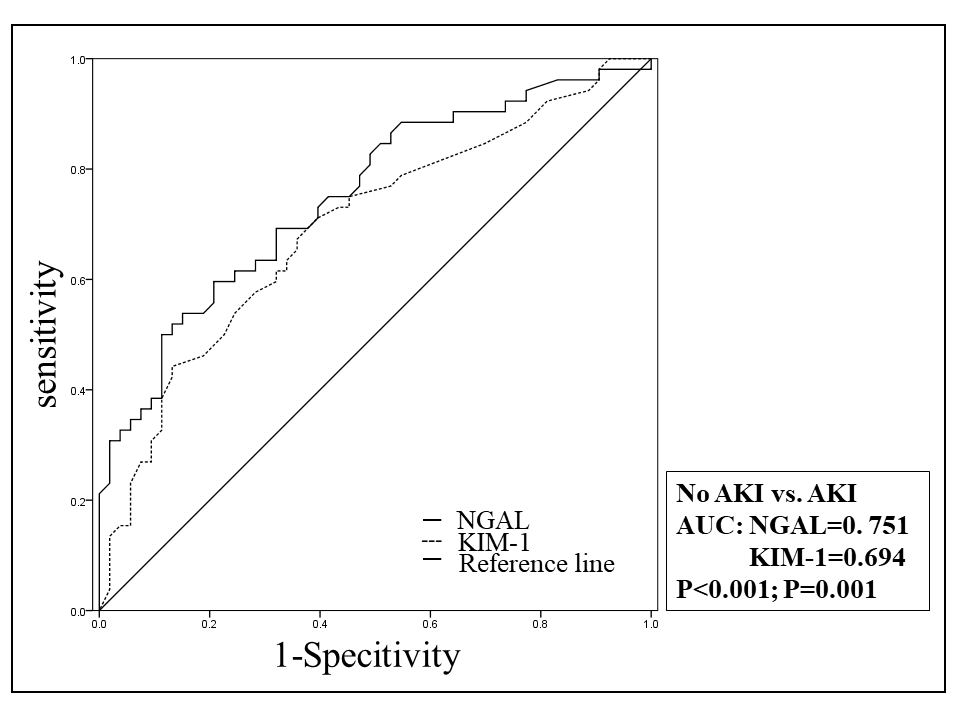


**Supplementary Figure 5.** Diagnostic effectiveness of urinary NGAL-1 and KIM-1 on the occurrence of AKI by ROC curve analysis.
